# Investigation of a Coupled Arrhenius-Type/Rossard Equation of AH36 Material

**DOI:** 10.3390/ma10040407

**Published:** 2017-04-13

**Authors:** Qin Qin, Ming-Liang Tian, Peng Zhang

**Affiliations:** 1 School of Mechanical Engineering, University of Science and Technology Beijing, Beijing 100083, China; tml817817@163.com; 2 The Tenth System Design Department of the Tenth Research Academy, Guiyang 550009, China; thmily@yeah.net

**Keywords:** high temperature tensile test, constitutive equation, flow stress, coupled equation

## Abstract

High-temperature tensile testing of AH36 material in a wide range of temperatures (1173–1573 K) and strain rates (10^−4^–10^−2^ s^−1^) has been obtained by using a Gleeble system. These experimental stress-strain data have been adopted to develop the constitutive equation. The constitutive equation of AH36 material was suggested based on the modified Arrhenius-type equation and the modified Rossard equation respectively. The results indicate that the constitutive equation is strongly influenced by temperature and strain, especially strain. Moreover, there is a good agreement between the predicted data of the modified Arrhenius-type equation and the experimental results when the strain is greater than 0.02. There is also good agreement between the predicted data of the Rossard equation and the experimental results when the strain is less than 0.02. Therefore, a coupled equation where the modified Arrhenius-type equation and Rossard equation are combined has been proposed to describe the constitutive equation of AH36 material according to the different strain values in order to improve the accuracy. The correlation coefficient between the computed and experimental flow stress data was 0.998. The minimum value of the average absolute relative error shows the high accuracy of the coupled equation compared with the two modified equations.

## 1. Introduction

AH36 is a typical product of the high-strength hull structural steel plates and is widely used in the shipbuilding industry. Quality standards of continuous casting plate for AH36 are strictly developed to meet the performance requirements. In order to improve the reasonable continuous casting process parameters, the constitutive equation of the material should be obtained to describe the relationship between flow stress, strain, strain rate, time at elevated temperatures, and other factors during the deformation process. However, this constitutive equation under high temperature is very complicated because it is affected by temperature, strain rate, and many parameters. Moreover, the deformation process also involves hardening and material dynamic softening mechanisms, such as dynamic recovery and dynamic recrystallization [1,2,3,4]. The comprehensive range of temperatures and large fluctuations of strain rates would exist under some deformation conditions. Therefore, it is a difficult task to reasonably determine the constitutive equation of a material because of the highly nonlinear behavior of this equation.

Many scholars have systematically studied the constitutive equations of different metals by using experimental methods. The exponent of temperature was proposed to describe the influence of the temperature on the flow stress by upsetting the specimens at different temperatures [5]. Litonski [6] improved the exponent of temperature to describe the relationship between the temperature and flow stress. However, the variation of the hardening coefficient and strain rate coefficient with temperature was ignored in these models. The strain hardening coefficient and strain rate coefficient, as functions of temperature, were introduced into the constitutive equation by Klepaczko [7] to improve the accuracy. With the wide acceptance of the Garofalo equation [8], the activation energy into the Arrhenius-type equation was suggested by Sellars and Tegart [9] based on the Garofalo equation. In these three Arrhenius-type equations, researchers believe that the sine hyperbolic-type of the Arrhenius equation was better than the power law of the Arrhenius equation [10]. The original sine hyperbolic constitutive equation has been modified many times to reasonably depict the deformation process for various alloys at elevated temperature due to ignoring the effects of strain and strain hardening on the deformation of materials. A strain-dependent parameter was introduced into the sine hyperbolic constitutive equation to represent the hot deformation process for wrought magnesium alloy Mg–Al4–Zn1 over a wide range of temperatures (523–773 K) and strain rates (0.01–100 s^−1^) [11]. The hyperbolic-sine equation was revised by Lin et al. [12] with the compensation of strain and strain rate to make a better prediction of the flow behaviors of 42CrMo steel at high temperatures from 1123 to 1423 K and strain rates from 0.01 to 50 s^−1^. The strain-compensated hyperbolic-sine equation was implemented to describe the hot deformation process for various materials by many researchers. Mandal [13] incorporated both the strain and strain rate compensation into the hyperbolic-sine equation to establish constitutive equation in a Ti-austenitic stainless steel at temperatures of 1123–1523 K and strain rates of 10^−3^–10^2^ s^–1^; Cai et al. [14] performed the constitutive analysis of Ti–6Al–4V alloy in a wide range of temperatures (1073–1323 K) and strain rates (0.0005–1 s^−1^) by using the modified hyperbolic-sine equation. Lin et al. [15] employed the strain-compensated equation to investigate the flow behaviors of hot deformation in 2124–T851 aluminum alloy over a wide range of temperatures (653–743 K) and strain rates (0.01–10 s^–1^); Li et al. [16] developed the strain-compensated equation to describe the hot deformation behaviors of 7050 aluminum alloy accurately in the temperature range of 573–723 K and strain rates of 0.001–1 s^−1^; Cai et al. [17] proposed the strain-dependent equation of BFe10-1-2 cupronickel alloy in a wide range of temperatures (1023–1273 K) and strain rates (0.001–10 s^−1^). In addition, the Backofen equation [18] was developed to depict the superplastic phenomenon. However, the predictability of this equation is relatively low because of ignoring the effect of strain on flow stress. Introducing strain and strain hardening into the Backofen equation was done by Fields [19] and Rossard [20] to improve the predictability. Some methods were presented to evaluate the values of strain-rate sensitive index and the strain hardening index [21,22]. Then the Rossard equation has been employed by some researchers to represent the hot deforming behaviors of magnesium alloy and aluminum alloy at a wide range of temperatures and strains [23,24]. The stress relaxation index and time parameter were introduced into the Rossard equation by Song [25] to describe the hot deformation process. The Rossard equation modified with the incorporation of peak strain and the consideration of temperature dependencies of the strain rate sensitivity and the stress coefficient was found to be appropriate for the prediction of flow stress [26]. The flow stress of dynamic recovery and dynamic recrystallization were assessed using different equations according to the critical strain for occurring dynamic recrystallization [27]. The two-stage equations were employed to consider the work softening behavior induced by dynamic recovery and dynamic recrystallization at temperatures of 1173–1473 K and strain rates of 1, 0.1, and 0.01 s^−1^ [28]. Haghdadi [29] has used the combined Estrin-Mecking and Avrami equations to describe the hot deformation behavior of LDX2101 with strain rates from 0.01 to 50 s^−1^ and temperatures from 1173 to 1523 K. However, the integrated constitutive equation was adopted to describe the relations between flow stress, temperature, strain and strain rate during all of the above models despite temperature and strain rates having a large variation range. The integrated constitutive equation cannot fully meet the investigation needs of continuous casting research. The reason is that this process is involved in across a comprehensive range of temperatures and large fluctuation of strain rates. Therefore, the coupled constitutive equation is suggested to meet the research needs of continuous casting.

Work by the authors of this paper not only focuses on the relationship between flow stress, strain, strain rate, and temperature at different segments of the hot deformation process for AH36 material, but it also aims to understand the coupled constitutive equation using experimental measurements. Therefore, the tensile deformation tests with solidified-type thermal histories are executed in the wide range of strain rates (10^−4^−10^−2^ s^−1^) and temperatures (1173–1573 K) by using the Gleeble-3500 system. Moreover, the modified Arrhenius-type equation and Rossard equation have been proposed in combination to describe the constitutive equation of AH36 material according to the different strain values. The Rossard equation has been used when the strain is less than 0.02 and the modified Arrhenius-type equation has been used when the strain is greater than 0.02 for improving the accuracy.

## 2. Experiment and Results

**Figure 1:**
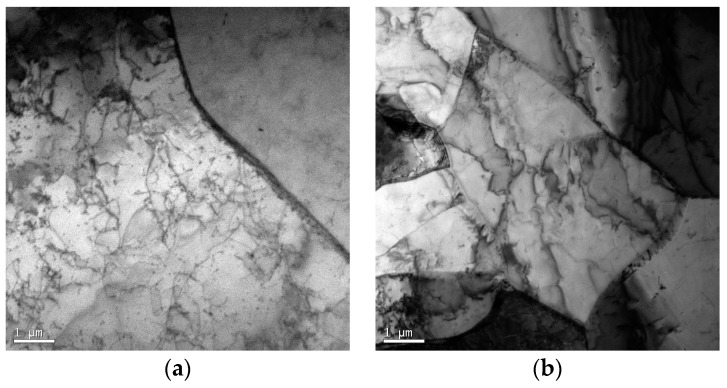
The initial microstructure of AH36 material by using Tecnai G2 F20.

**Figure 2:**
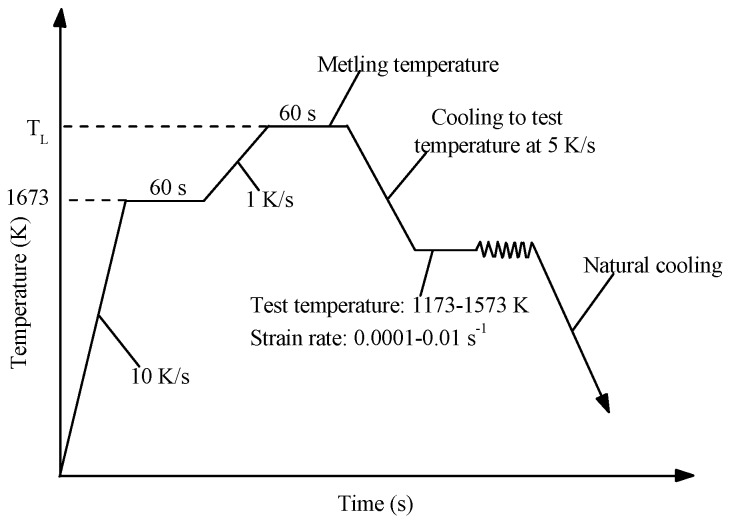
Schematic of the thermal histories for tensile test.

The AH36 material as the experimental sample was used for hot tensile testing in the present study. The chemical composition of the AH36 material is shown in Table 1. The initial microstructure of AH36 by using Tecnai G2 F20 is shown in Figure 1. As can be seen in Figure 1a, a large number of dislocations are distributed in the regions of martensite. There are also some ferrites, polygonal in shape, as shown in Figure 1b. The quasi-isothermal tensile tests were performed by employing a Gleeble-3500 materials simulator at temperatures ranging from 1173 to 1573 K at an interval of 100 K, with constant strain rates of 10^−4^, 10^−3^, and 10^−2^ s^−1^. All standard cylindrical specimens for tension testing machined from a continuous casting slab were 10 mm in diameter and 120 mm in height, with screws at both ends. The longitudinal direction of the specimen was normal to both the casting direction and the columnar dendrite growth direction. The center of the specimen was installed with quartz glass tube with a length of 30 mm and inner-diameter of 10.4 mm to support the melting zone of about 10 mm in length and prevent heat loss at the uniform temperature area. A R-type (Pt–Pt13%Rh) thermocouple was used to control the temperature at the mid-span surface of the specimen during testing. In order to block oxidation of the specimen and minimize the heat convection where it might induce a radial thermal gradient, the hot tensile tests were conducted under vacuum. As can be seen in Figure 2, all of the specimens were rapidly heated to 1673 K with a heating rate of 10 K/s, held for 60 s to make it fully austenitizing, and then reheated to the melting temperature with a slower heating rate of 1 K/s. After being held for 60 s for melting, the specimens were cooled down to different testing temperatures with a cooling rate of 5 K/s. Finally, tensile tests were carried out with the defined strain rate at the testing temperature. The specimens were finally cooled down by the natural cooling.

Some scholars believe that the strain measurement is relative accuracy by using the C-gauge because the instantaneous cross-sectional area as well as the strain in the lateral direction is clamped can be monitored and recorded [30]. But the specimens have been heated to the melting temperature and resolidified in this study to simulate the continuous casting process. In order to ensure safety of the experimenters and the Gleeble system, an equipment of the quartz glass tube was installed in the center of the specimen to support the melting zone and prevent the leakage of liquid metal and heat loss at the uniform temperature area. It is a difficult task that the strain measurement can be obtained by using the C-gauge when a quartz glass tube is installed in the center of the specimen. Therefore, the L-gauge method has been chosen to measure the strain along the axial direction in this study during the quasi-isothermal tensile test. The gauge lengths are approximate 10 mm in the central region of the specimens. In addition, the tensile tests of the specimen under the high temperature and low strain rate were carried out to verify the difference of tensile testing results respectively by using the C-gauge and the L-gauge. The temperatures were designed to 1573 K and 1473 K with a strain rate of 10^−3^ s^−1^ because these temperatures close to the melting temperature of AH36 in the thermal histories and the quartz tubes were avoided. And the C-gauge and L-gauge methods were adopted to measure the strain. The maximum values of the absolute error between the experimental data from the C-gauge and the L-gauge are 0.39% and 0.36%, respectively, when the temperatures are 1473 and 1573 K. And the average values of the absolute error are 0.23% and 0.21%, respectively, when the temperatures are 1473 and 1573 K. Therefore, the L-gauge method was adopted to measure the strain under the continuous casting conditions of high temperature and low strain rate because of the small deviation of the experimental data respectively obtained by the C-gauge and the L-gauge.

**Figure 3:**
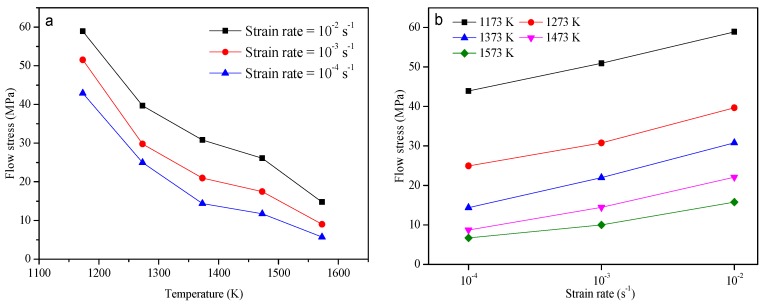
Flow curves of AH36 material with a strain level of 0.04 at various (**a**) strain rates (10^−4^–10^−2^ s^−1^) and (**b**) temperatures (1173–1573 K).

Based on the isothermal hot tension test results, the flow curves of AH36 material at various deformation conditions are shown in Figure 3. It can be observed that the flow stress decreases with the increase of temperature and the decrease of strain rate, and the trend of the evolution of flow stress during the deformation process is similar to that of many metals researched previously. Exemplarily for the test at the strain rate of 10^−2^ s^−1^ in Figure 3a, the absolute values of the slopes at each segment of temperatures are 0.19, 0.09, 0.05 and 0.11, respectively. As can be seen in Figure 3b, the slopes of flow curves at each segment of strain rates are 8444.3 and 981.7 at 1373 K, as well as 3681.5 and 639.2 at 1573 K. The slopes of the lines in the *σ*-T plot are changing in different segments of temperatures, and the difference of the slope of flow stress vs. strain rate in various segments of strain rates is evident. Thus the sensitivity of flow stress to temperature and strain rate is significant. Furthermore, the values of flow stress decrease by 44.15 MPa, 42.53 MPa, and 37.21 MPa, respectively, when the strain rates are maintained at 10^−2^, 10^−3^, and 10^−4^ s^−1^ with the temperature increasing from 1173 to 1573 K at a strain of 0.04. The large variation of flow stress is evident at different strain rates with the change of temperature.

## 3. Discussion

### 3.1. Modified Arrhenius-Type Equation

The strain and strain hardening are introduced into the hyperbolic law in an Arrhenius-type of equation to modify the model by taking into account the Hollomon equation about flow stress vs. strain at low strain conditions. The formulations are expressed as follows:

(1) $$\left\{ \begin{array}{l} \dot{\varepsilon }=Cf\left(h\right)\exp\left(-\frac{Q}{RT}\right)\\ \sigma =h\varepsilon^{m} \end{array}\right.$$

where

$$f\left(h\right)=\left\{ \begin{array}{ll} h^{k_{1}} & \gamma h<0.8\\ \exp\left(\lambda h\right) & \gamma h>1.2\\ {\left[\sin h\left(\gamma h\right)\right]}^{k} & for\;all\;h \end{array}\right.$$

Here, $\dot{\varepsilon }$ is the strain rate, s^−1^; *ε* is the strain; Q is the activation energy, J mol^−1^; *R* is the gas constant, 8.314 J mol^−1^ K^−1^; *T* is the absolute temperature, K; *C*, *k*_1_, *k*, *λ*, and *γ* are the material constants; *m* is the strain hardening exponent; and *h* is the strength coefficient.

The influence of strain rate and temperature on flow stress could be described by Zener-Holloman parameter in an exponent-type equation:

(2) $$Z=\dot{\varepsilon }\exp\left(\frac{Q}{RT}\right)$$

The relationship between flow stress and the Zener-Holloman parameter could be defined taking into consideration Equations (1) and (2):

(3) $$Z=C{\left[\sin h\left(\gamma \frac{\sigma }{\varepsilon^{m}}\right)\right]}^{k}$$

**Figure 4:**
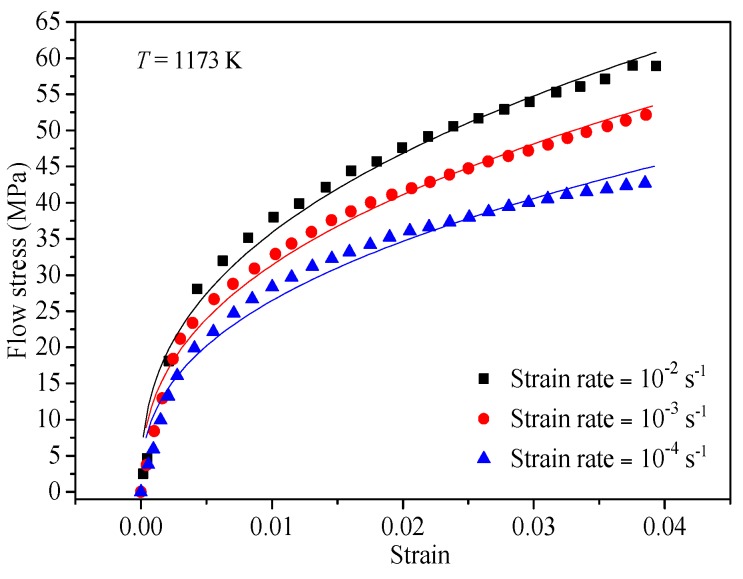
Evaluating the value of *m* by fitting the *σ* vs. *ε* data at a temperature of 1173 K.

The material constants of the modified Arrhenius-type equation could be evaluated respectively using the experimental data from hot tensile tests at various deformation conditions. In the present study, the temperature of 1173 K was taken as an example to introduce the evaluation procedure of material constants as follows:

The value of *m* can be achieved by fitting the true stress-strain data under various deformation strain rates at a temperature of 1173 K considering the Hollomon equation. As can be seen in Figure 4, the value of *m* can be found by taking the mean value at different strain rates ignoring the trivial variation in the obtained strain hardening exponent.

As shown in Equation (1), *f*(*h*) is substituted by the power law when the value of *γh* is less than 0.8, and by the exponential law when *γh* is greater than 1.2. Then applying $\frac{\sigma }{\varepsilon^{m}}$ in place of *h*, Equations (4) and (5) can be obtained as follows:

(4) $$\dot{\varepsilon }=C_{1}{\left(\frac{\sigma }{\varepsilon^{m}}\right)}^{k_{1}}\exp\left(-\frac{Q}{RT}\right)$$

(5) $$\dot{\varepsilon }=C_{2}\exp\left(\lambda \frac{\sigma }{\varepsilon^{m}}\right)\exp\left(-\frac{Q}{RT}\right)$$

where *C*_1_ and *C*_2_ are the material constants.

The logarithm of both sides of Equations (4) and (5) were taken. Then the Equations (6) and (7) can be achieved as follows:

(6) $$\ln\dot{\varepsilon }=\ln C_{1}+k_{1}\ln\left(\frac{\sigma }{\varepsilon^{m}}\right)-\frac{Q}{RT}$$

(7) $$\ln\dot{\varepsilon }=\ln C_{2}+\lambda \left(\frac{\sigma }{\varepsilon^{m}}\right)-\frac{Q}{RT}$$

**Figure 5:**
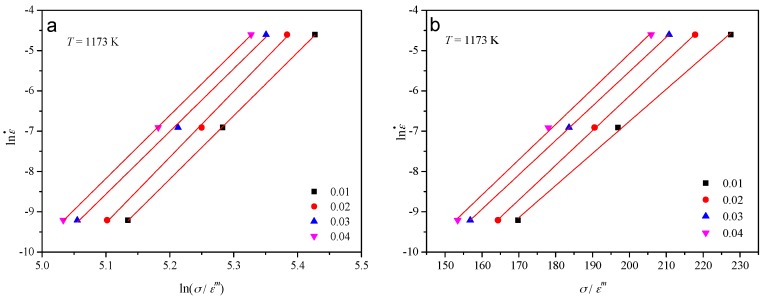
Evaluating the value of (**a**) *k*_1_ by plotting $\ln\dot{\varepsilon }$ vs. ln(*σ*/*ε^m^*) and (**b**) *λ* by plotting $\ln\dot{\varepsilon }$ vs. (*σ*/*ε^m^*).

The experimental data of flow stress and corresponding strain rate under various strains at the temperature of 1173 K were substituted into Equations (6) and (7). The values of *k*_1_ and *λ* can be calculated from the slope of the lines in the $\ln\dot{\varepsilon }$ vs. ln(*σ*/*ε^m^*) and $\ln\dot{\varepsilon }$ vs. (*σ*/*ε^m^*) plots respectively. As can be seen in Figure 5, the relationship between strain rate and the strength coefficient can be approximated by a group of parallel straight lines. The mean values of the slopes in the two diagrams were taken as the values of *k*_1_ and *λ* respectively. Then the corresponding value of *γ* = *λ*/*k*_1_ could be determined.

For all the strength coefficient level, the Equation (1) can be represented with the $\frac{\sigma }{\varepsilon^{m}}$ in place of *h*, as follows:

(8) $$\dot{\varepsilon }=C{\left[\sin h\left(\gamma \frac{\sigma }{\varepsilon^{m}}\right)\right]}^{k}\exp\left(-\frac{Q}{RT}\right)$$

(9) $$\ln\dot{\varepsilon }=\ln C+k\ln\left[\sin h\left(\gamma \frac{\sigma }{\varepsilon^{m}}\right)\right]-\frac{Q}{RT}$$

**Figure 6:**
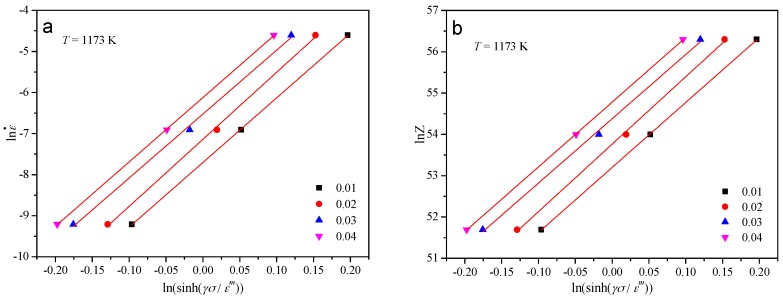
Evaluating the value of (**a**) *k* by plotting $\ln\dot{\varepsilon }$ vs. ln(sinh(*γσ*/*ε^m^*)) at various strains and (**b**) ln*C* by plotting ln*Z* vs. ln(sinh(*γσ*/*ε^m^*)) at various strains.

The tensile test results under various strains at the temperature of 1173 K were employed into the Equation (9). Then the relationship between $\ln\dot{\varepsilon }$ and ln(sinh(*γσ*/*ε^m^*)) can be obtained. As shown in Figure 6a, the value of *k* can be derived from the mean value of the slopes of the lines in the $\ln\dot{\varepsilon }$ −ln(sinh(*γσ*/*ε^m^*)) plot.

**Figure 7:**
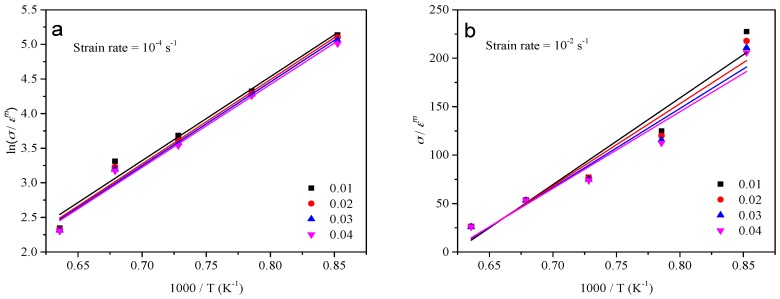
Evaluating the value of (**a**) *m*_1_ by plotting ln(*σ*/*ε^m^*) vs. 1000/*T* at a strain rate of 10^−4^ s^−1^ and (**b**) *m*_2_ by plotting *σ*/*ε^m^* vs. 1000/*T* at a strain rate of 10^−2^ s^−1^.

The activation energy Q can be evaluated with Equation (10) by differentiating Equation (6) when the value of *γh* is relatively low. Instead, Q can be described by Equation (11) by calculating the partial differential of Equation (7) at the high value of *γh*. As can be seen in Figure 7a, the relationship between ln(*σ*/*ε^m^*) and 1000/*T* can be described by a group of parallel lines, and the slight variation in the slope of the lines in Figure 7b could be ignored, attributed to scattering in the experimental data points. The values of *m*_1_ and *m*_2_ can be, respectively, gained by taking the mean value of slopes in Figure 7a,b, then substituting the *m*_1_ and *m*_2_ into Equations (10) and (11), the value of the activation energy, as a constant, can be obtained by averaging the values of Q at 10^−2^ and 10^−4^ s^−1^ due to the slight discrepancy between them.

(10) $$\left\{ \begin{array}{l} Q=1000\cdot Rk_{1}m_{1}\\ m_{1}=\frac{\partial \ln\left(\sigma /\varepsilon^{m}\right)}{\partial \left(1000/T\right)} \end{array}\right.$$

(11) $$\left\{ \begin{array}{l} Q=1000\cdot R\lambda m_{2}\\ m_{2}=\frac{\partial \left(\sigma /\varepsilon^{m}\right)}{\partial \left(1000/T\right)} \end{array}\right.$$

Equation (12) can be obtained by taking the logarithm of both sides of Equation (3):

(12) $$\ln Z=k\ln\left[\sin h\left(\gamma \frac{\sigma }{\varepsilon^{m}}\right)\right]+\ln C$$

The experimental data under different strains at the temperature of 1173 K were substituted into the Equation (12). The correlation between lnZ and ln(sinh(*γσ*/*ε^m^*)) can be achieved. As shown in Figure 6b, the material constant *C* can be evaluated from the intercept of ln*Z* − ln(sinh(*γσ*/*ε^m^*)).

**Figure 8:**
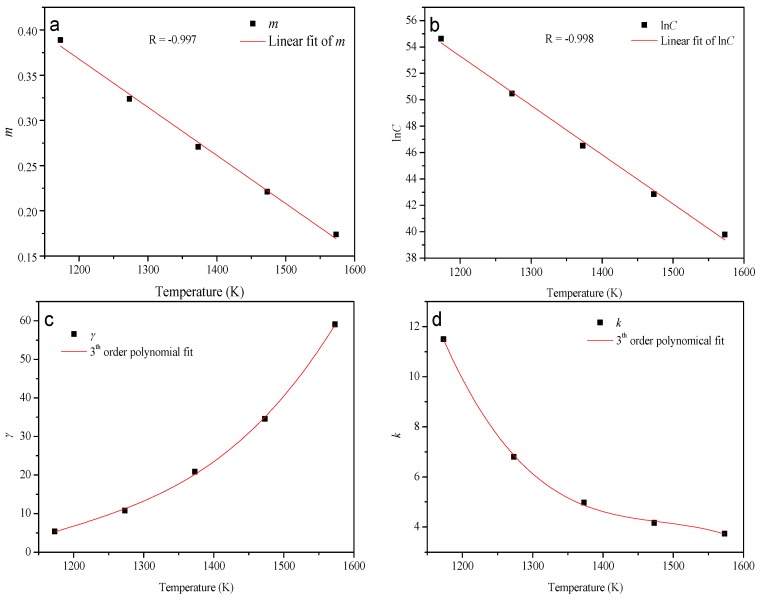
Variation of (**a**) *m*; (**b**) ln*C*; (**c**) *γ*; and (**d**) *k* with the temperature.

The material constants of *m*, *γ*, *k*, and *C* at the temperatures of 1273–1573 K in the range of strain rates 10^−4^–10^−2^ s^−1^ can be, respectively, evaluated in the ways represented above. The dependency of material constants on temperature can be derived by assuming that those material constants are the polynomial functions of temperature. The values of those material constants obtained at different temperatures were then employed to fit the polynomial. As can be seen in Figure 8, all of the polynomials are found to represent the influence of temperature on the material constants with a good correlation and generalization. The formulations are expressed as follows:

(13) $$\left\{ \begin{array}{l} m=-5.0993\times 10^{-4}T+0.9757\\ \gamma =2.619937\times 10^{-10}T^{3}-7.888465\times 10^{-7}T^{2}+8.059853\times 10^{-4}T-0.277340\\ k=-1.475917\times 10^{-7}T^{3}+6.787172\times 10^{-4}T^{2}-1.042072T+538.1333\\ \ln C=-0.0353T+95.4981\\ Q=619.171\times 10^{3}\;J\cdot mol^{-1} \end{array}\right.$$

With the definition of the hyperbolic law and the Zener-Holloman parameter, the modified Arrhenius-type equation relating flow stress, strain rate, strain, and temperature can be described in the following form:

(14) $$\sigma =\frac{1}{\gamma }\ln\left\{{\left(\frac{\dot{\varepsilon }\exp\left(\frac{Q}{RT}\right)}{C}\right)}^{1/k}+{\left[{\left(\frac{\dot{\varepsilon }\exp\left(\frac{Q}{RT}\right)}{C}\right)}^{2/k}+1\right]}^{1/2}\right\}\varepsilon^{m}$$

### 3.2. Modified Rossard Equation

The variation of the hardening coefficient and strain rate coefficient with the deformation conditions was introduced into the Rossard equation to improve the accuracy. The modified Rossard equation can be represented as follows:

(15) $$\sigma =K\varepsilon^{p}{\dot{\varepsilon }}^{q}$$

where *K* is the material constant; *p* is the strain-hardening index, and *q* is the strain-rate sensitive index. All of them are a function of the deformation conditions.

The Equation (16) can be obtained by taking the logarithm of both sides of Equation (15):

(16) $$\ln\sigma =p\ln\varepsilon +q\ln\dot{\varepsilon }+\ln K$$

**Figure 9:**
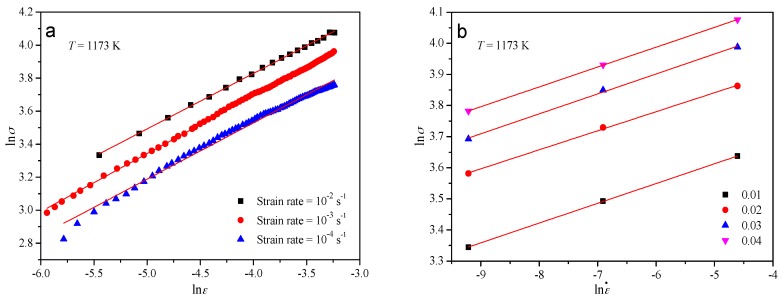
Evaluating the value of (**a**) *p* by plotting ln*σ* vs. ln*ε* and (**b**) *q* by plotting ln*σ* vs. $\ln\dot{\varepsilon }$.

The experimental data from hot tensile tests at various deformation conditions have been employed to derive the material constant *K*, the strain-hardening index *p* and the strain-rate sensitive index *q*. The evaluation procedure of material constants was represented as follows:

The tensile test results under various strain rates at the temperature of 1173 K were substituted into Equation (16). The strain-hardening index *p* and the strain-rate sensitive index *q* can be calculated by plotting ln*σ* vs. ln*ε* and ln*σ* vs. $\ln\dot{\varepsilon }$, respectively. As can be seen in Figure 9a, the values of *p* under the strain rates of 10^−2^, 10^−3^, and 10^−4^ s^−1^ were, respectively, obtained at a temperature of 1173 K. The relationship between ln*σ* and $\ln\dot{\varepsilon }$ at different strains can be described by a group of parallel lines as shown in Figure 9b. The value of *q* can be gained at 1173 K by averaging the slopes of the lines. The values of *p* and *q* under other thermo-mechanical conditions can be obtained in the ways described above. 

Equation (17) can be achieved by rearranging Equation (15) represented as follows:

(17) $$K=\frac{\sigma }{\varepsilon^{p}{\dot{\varepsilon }}^{q}}$$

**Figure 10:**
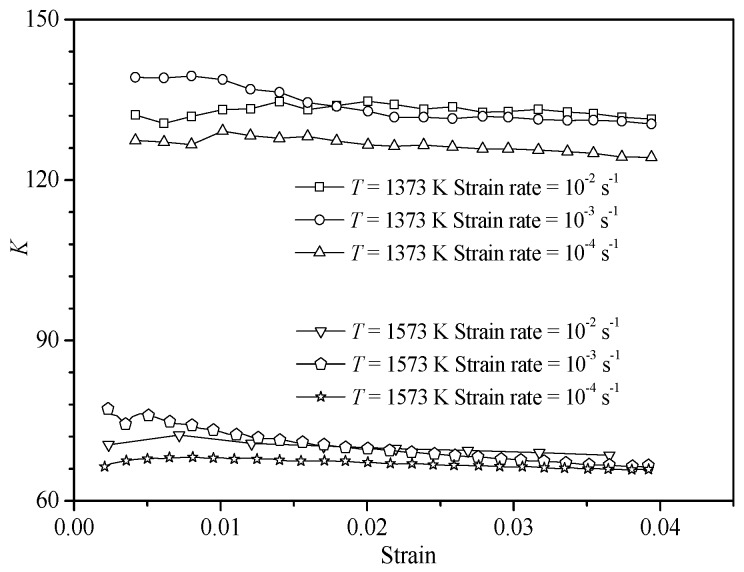
Variation of *K* with strain at temperatures of 1373 and 1573 K under different strain rates.

The experimental data from hot tensile tests at temperatures of 1373 and 1573 K under the entire strain rate range were employed to obtain the material constant *K*. As can be seen in Figure 10, the variation of the value *K* with strain is slight at the same temperature. Further, the material constant *K* consequently varies in a very small range at a certain deformation temperature. The small variation could be attributed to scattering in the experimental data points. Then the values of *K* were, respectively, obtained at the temperatures of 1373 and 1573 K by taking the mean value. The value of *K* at other temperatures can be calculated in the same way.

**Figure 11:**
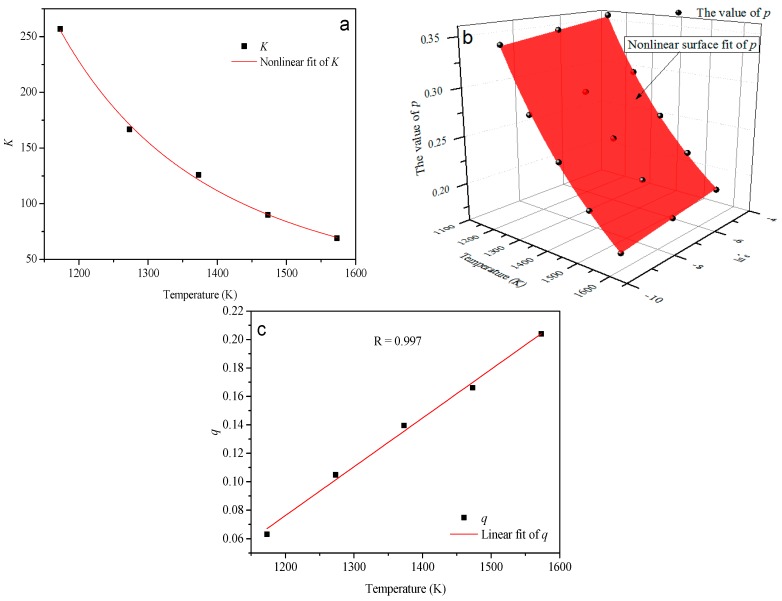
Variation of (**a**) *K*; (**b**) *p*; and (**c**) *q* with the thermo-mechanical conditions.

The values of *K*, *p* and *q* obtained at different thermo-mechanical conditions were then applied to derive the relationship between the material constants and deformation conditions. As shown in Figure 11, all of the formulations show a good correlation and generalization. The expressions of those formulations are indicated as follows:

(18) $$\left\{ \begin{array}{l} K=1.678\times \exp\left(\frac{5892.778}{T}\right)\\ p=-0.01+0.0036\ln\dot{\varepsilon }+\frac{526548}{T^{2}}\\ q=3.42875\times 10^{-4}T-0.33325 \end{array}\right.$$

After the material constants of the modified Rossard equation were determined, the modified Rossard equation can be expressed as:

(19) $$\sigma =g\exp\left(\frac{h}{T}\right)\varepsilon^{\left(a+b\ln\dot{\varepsilon }+\frac{c}{T^{2}}\right)}{\dot{\varepsilon }}^{dT+e}$$

where, *a*, *b*, *c*, *d*, *e*, *g* and *h* are the material constants. For AH36 material, the values of *a* = −0.01, *b* = 0.0036, *c* = 526,548, *d* = 3.42875 × 10^−4^, *e* = −0.33325, *g* = 1.678, and *h* = 5892.778 have been obtained.

### 3.3. Verification of the Modified Arrhenius-Type Equation and Rossard Equation

**Figure 12:**
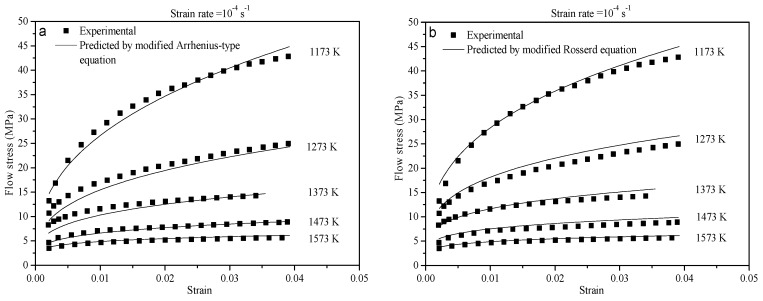
Comparison between the experimental and predicted flow stress from (**a**) the modified Arrhenius-type equation and (**b**) the modified Rossard equation at a strain rate of 10^−4^ s^−1^.

The modified Arrhenius-type equation and Rossard equation have been verified by comparing the experimental data with predicted values. The typical experimental data and predicted values are shown in Figure 12 when the strain rate is 10^−4^ s^−1^. As can be seen in Figure 12, the average absolute relative error between the predicted data of the modified Arrhenius-type equation and the experimental results is 7.56% when the strain is less than 0.02. This error reaches 4.34% when the strain is greater than 0.02. The average absolute relative error between the predicted data of the Rossard equation and the experimental results is 6.99% when the strain is greater than 0.02. This error reaches 1.78% when the strain is less than 0.02. The errors between the predicted data and the experimental results under the strain rate of 10^−2^ and 10^−3^ s^−1^ are listed in Table 2. As can be seen in Table 2, the law of flow curves at the different strain rate indicates almost the same rule. This rule is that there is a good agreement between the predicted data of the modified Arrhenius-type equation and the experimental results when the strain is greater than 0.02. However, there is good agreement between the predicted data of the Rossard equation and the experimental results when the strain is less than 0.02. The reason is possibly that the internal dislocation density increases and strain hardening is severe at the beginning of the deformation process. A better prediction of the modified Rossard equation has been observed by taking into account the two hardening factors of the strain-hardening index and strain-rate sensitive index. The dynamic recovery is strengthened resulting in the reduction of work hardening when the strain increases. The modified Arrhenius-type equation is, consequently, found to be better to describe the flow stress.

Therefore, the modified Arrhenius-type equation and the Rossard equation have been adopted in combination to describe the constitutive equation of AH36 material for improving the accuracy according to the different strain values. The Rossard equation has been used when the strain is less than 0.02 and the modified Arrhenius-type equation has been used when the strain is greater than 0.02 in the present study.

### 3.4. Establishment of the Coupled Constitutive Equation

Based on the analysis of the suitable scope of the two modified equations, the predictability of the modified Arrhenius-type equation is preferred for relatively high-strain conditions (*ε* > 0.02), whereas the modified Rossard equation is suitable for low-strain conditions when the strain is less than 0.02. Thus, the constitutive model of AH36 material at high temperature can be developed with the two modified equations. The strain of 0.002 has been taken as the yield criterion for there is no distinct yield phenomenon at elevated temperatures and low strain rates. Then the deformation process was distributed into the regions of higher work hardening in the range of strains 0.002–0.02 and lower work hardening in the range of strains 0.02–0.04. The constitutive analysis can be depicted with the modified Rossard equation at the regions of higher work hardening, and the modified Arrhenius-type equation at the regions of lower work hardening. As can be seen in Equation (20), the constitutive equations at different domains of strains have been constituted.

(20) $$\left\{ \begin{array}{ll} \sigma =g\exp\left(\frac{h}{T}\right)\varepsilon^{\left(a+b\ln\dot{\varepsilon }+\frac{c}{T^{2}}\right)}{\dot{\varepsilon }}^{dT+e} & 0.002<\varepsilon <0.02\\ \sigma =\frac{1}{\gamma }\ln\left\{{\left(\frac{\dot{\varepsilon }\exp\left(\frac{Q}{RT}\right)}{C}\right)}^{1/k}+{\left[{\left(\frac{\dot{\varepsilon }\exp\left(\frac{Q}{RT}\right)}{C}\right)}^{2/k}+1\right]}^{1/2}\right\}\varepsilon^{m} & 0.02<\varepsilon <0.04 \end{array}\right.$$

where all of the material constants can be calculated from Equations (14) and (19). 

### 3.5. Verification of the Coupled Constitutive Equation

**Figure 13:**
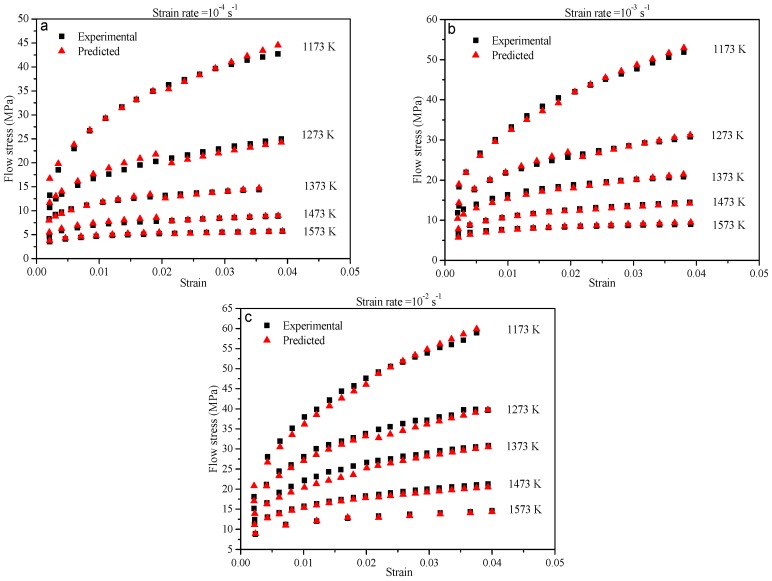
Comparison between the experimental and predicted flow stress from the coupled constitutive equation at strain rates of (**a**) 10^−4^ s^−1^; (**b**) 10^−3^ s^−1^; and (**c**) 10^−2^ s^−1^.

The predictive ability of the coupled constitutive equation was appraised with the comparison between the experimental and predicted data. As can be seen in Figure 13, the computed flow stress from the coupled constitutive equation could track the experimental data well throughout the entire strain rates. The calculated values of average absolute relative errors by the coupled model are 3.38%, 2.56%, and 3.42% at the strain rates of 10^−4^, 10^−3^, and 10^−2^ s^−1^, respectively. All of the values of the errors are within the range of allowable experimental conditions.

Exemplarily for the test at the strain rate of 10^−4^ s^−1^, the average absolute relative errors at low-strain conditions and high-strain conditions are, respectively, found to be 1.78% and 4.34% along the entire temperature range. Compared with the modified Arrhenius-type equations, the decrease of the computed average absolute relative error by the coupled equation is apparent under low-strain conditions. The value of the error has fallen from 7.56% to 1.78% (a decrease of 76.5%). There is also an evident decrease in the calculated average absolute relative error by Equation (20) under high-strain conditions confronted with the modified Rossard equation. The value of the error has caused a 37.9% decrease. Thus, the accuracy of the coupled constitutive equation has been greatly improved.

The predictability of the coupled constitutive equation has been validated with the statistical analysis of the correlation coefficient (R) and average absolute relative error (AARE). The correlation coefficient provides information about the strength of the linear relationship between the experimental and predicted data [31]. However, sometimes a higher value of R may not consequentially indicate a better performance of the model because of the model may be biased towards higher or lower values. The AARE is an unbiased statistical parameter for demonstrating the accuracy of a model because the value of AARE is calculated through a term-by-term comparison of the relative error [32]. The formulas of them are expressed as in Equations (21) and (22).

(21) $$R=\frac{\sum_{i=1}^{N}\left(E_{i}-\bar{E}\right)\left(P_{i}-\bar{P}\right)}{\sqrt{\sum_{i=1}^{N}{\left(E_{i}-\bar{E}\right)}^{2}\sum_{i=1}^{N}{\left(P_{i}-\bar{P}\right)}^{2}}}$$

(22) $$\mathrm{AARE}\left(\%\right)=\frac{1}{N}\sum_{i=1}^{N}\left|\frac{E_{i}-P_{i}}{E_{i}}\right|\times 100$$

where *E* is the experimental data and *P* is the predicted value calculated by the modified constitutive equation; $\bar{E}$ and $\bar{P}$ are the mean values of experimental and predicted results, respectively; and *N* is the total number of employed data points in the investigation.

**Figure 14:**
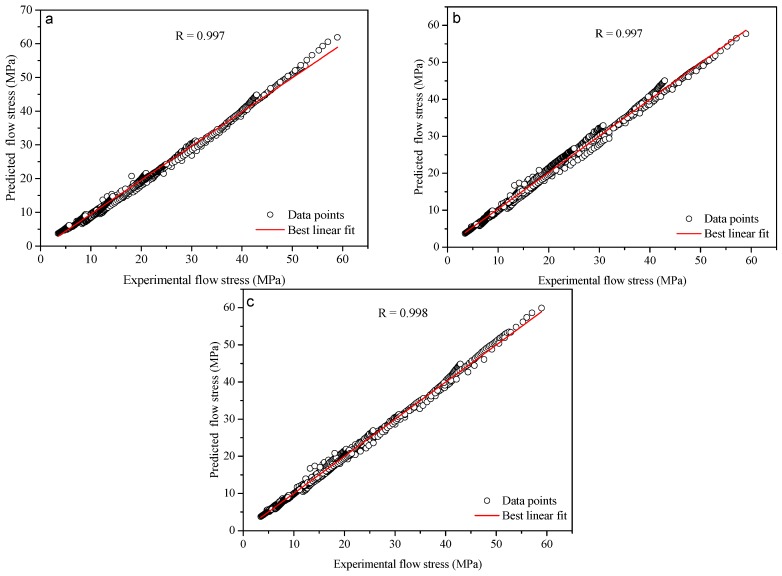
Correlation between the experimental and predicted flow stress data from (**a**) the modified Arrhenius-type equation; (**b**) the modified Rosserd equation; and (**c**) the coupled constitutive equation.

The correlation between the experimental and predicted flow stress data from the coupled constitutive equation is shown in Figure 14c. It can be seen that most of the data points lie close to the best regression line, and the correlation coefficient for the coupled model is 0.998. The best correlation between the experimental and predicted flow stress data was evident. The determined value of the AARE is 3.02%.

To further investigate the performance of the coupled constitutive equation, the comparison between the two modified equations and the coupled constitutive equation has been conducted under various deformation conditions. The determined values of the correlation coefficient calculated from the two modified equations and the coupled constitutive equation were, respectively, shown in Figure 14. It can be seen that the best correlation between the experimental and predicted flow stress from the coupled model is visible compared with the two modified equations. Further, the determined values of the AARE by the two modified equations are 5.64% and 4.48%, respectively. Both of them are greater than the value 3.02% by the coupled model. A good performance of the coupled constitutive equation is significant.

## 4. Conclusions

The coupled constitutive equation of AH36 material has been derived by performing hot tension tests in the temperature range of 1173–1573 K and strain rate range of 10^−4^–10^−2^ s^−1^. The conclusions obtained are as follows:

(1) The material properties of AH36 material are sensitive to the temperature and strain rate at hot deformation conditions, where flow stress decreases with the increase of temperature and the shrinkage of the strain rate. The value of flow stress respectively decreases by 44.15 MPa, 42.53 MPa, and 37.21 MPa when the strain rates are maintained at 10^−2^, 10^−3^, and 10^−4^ s^−1^ with the temperature increasing from 1173 to 1573 K at a strain of 0.04.
(2) The average absolute relative error between the predicted data of the modified Arrhenius-type equation and the experimental results is 7.56% when the strain is less than 0.02 at a strain rate of 10^−4^ s^−1^. This error reaches 4.34% when the strain is greater than 0.02. The laws of strain rates of 10^−2^ and 10^−3^ s^−1^ are also similar to that of 10^−4^ s^−1^. Thus, the predictability of the modified Arrhenius-type equation is preferred for relatively high-strain conditions.
(3) The average absolute relative error between the predicted data of the Rossard equation and the experimental results is 6.99% when the strain is greater than 0.02. This error reaches 1.78% when the strain is less than 0.02. The laws at strain rates of 10^−2^ and 10^−3^ s^−1^ are also similar to that of 10^−4^ s^−1^. Thus, the modified Rossard equation is suitable for low-strain conditions.
(4) The modified Arrhenius-type equation and Rossard equation have been adopted in combination to describe the constitutive equation of AH36 material for improving the accuracy according to the different strain value. The Rossard equation has been used when the strain is less than 0.02, and the modified Arrhenius-type equation when the strain is greater than 0.02. The correlation coefficient for the coupled model is 0.998. The determined value of the AARE is 3.02%, which shows good predictability of the coupled model. The best correlation and the minimum value of average absolute relative error of the coupled model show the high accuracy of the coupled model compared with the two modified equations.

## Figures and Tables

**Table 1:** Chemical composition of AH36 steel (in wt %).

Fe	C	Si	Mn	P	S	Als	Cr	Mo	Ni	Cu
98.251	0.157	0.2489	1.1132	0.0162	0.0044	0.0289	0.0375	0.0045	0.0177	0.0284

**Table 2:** The average absolute relative error between the predicted data and the experimental results under the strain rates of 10^−2^ and 10^−3^ s^−1^.

Strain Rate (s^−1^)	Modified Arrhenius-Type Equation		Modified Rossard Equation	
	*ε* < 0.02	*ε* > 0.02	*ε* < 0.02	*ε* > 0.02
10^−2^	5.02%	3.57%	3.28%	3.54%
10^−3^	5.90%	2.94%	2.01%	3.11%
